# Abdominal adipose tissue thickness measured using magnetic resonance imaging is associated with lumbar disc degeneration in a Chinese patient population

**DOI:** 10.18632/oncotarget.13255

**Published:** 2016-11-09

**Authors:** Lili Yang, Liangshan Mu, Kaiyu Huang, Tianyi Zhang, Zihan Mei, Wenrong Zeng, Jiawei He, Wei Chen, Xiaozheng Liu, Xinjian Ye, Zhihan Yan

**Affiliations:** ^1^ Radiology Department, The Second Affiliated Hospital and Yuying Children's Hospital of Wenzhou Medical University, Wenzhou, People's Republic of China; ^2^ China-USA Neuroimaging Research Institute of Wenzhou Medical University, Wenzhou, People's Republic of China; ^3^ Reproductive Medicine Center, The First Affiliated Hospital of Wenzhou Medical University, Wenzhou, People's Republic of China; ^4^ Wenzhou Medical University, Wenzhou, People's Republic of China

**Keywords:** intervertebral disc degeneration, low back pain, abdominal fat, magnetic resonance imaging, Gerotarget

## Abstract

The relationship between abdominal adiposity and disc degeneration remains largely uninvestigated. Here, we investigated the association between abdominal adipose tissue thickness and lumbar disc degeneration in a cross-sectional study of 2415 participants from The Second Affiliated Hospital of Wenzhou Medical University. All subjects were scanned with a 3T Magnetic Resonance Imaging system to evaluate the degree of lumbar disc degeneration. Multiple logistic regression analysis revealed that men in the highest quartiles for abdominal diameter (AD), sagittal diameter (SAD), and ventral subcutaneous thickness (VST) were at higher odds ratio for severe lumbar disc degeneration than men in the lowest quartiles. The adjusted model revealed that women in the highest quartiles for AD and SAD were also at higher odds ratio for severe lumbar disc degeneration than women in the lowest quartiles. Our results suggest that abdominal obesity might be one of underlying mechanisms of lumbar disc degeneration, and preventive strategies including weight control could be useful to reduce the incidence of lumbar disc degeneration. Prospective studies are needed to this confirm these results and to identify more deeper underlying mechanisms.

## INTRODUCTION

The proportion of the population that is overweight or obese has increased dramatically worldwide in recent decades, and obesity has become an important public health issue [1]. Obesity is linked to the development of many diseases, including metabolic syndromes, diabetes mellitus, cardiovascular diseases, and cancers [2]. Recent research has also indicated that being overweight or obese is strongly associated with disc degeneration [3, 4]; in particular, abdominal obesity has been implicated in lumbar disc degeneration [5], which is related to lower back pain (LBP) and diminished physical and social functioning [6, 7]. Several mechanisms, including mechanical overloading, metabolic syndromes, and systematic chronic inflammation, may account for this link [8, 9].

Body mass index (BMI) is widely used to measure body composition and classify patients as overweight or obese [3, 6, 10]. Another measurement, waist circumference (WC), is usually used to specifically evaluate abdominal obesity. However, both methods have obvious limitations; for example, BMI does not account for the distribution of body fat and muscle mass or their relative proportions [11], and is therefore skewed in individuals who are highly muscular or who have muscle wasting. However, magnetic resonance imaging (MRI) has proven useful for measuring adipose tissue in the body [12] and abdominal obesity [13]; the use of MRI rather than BMI would also improve investigations of the relationship between obesity and lumbar disk degeneration. MRI is also superior to WC or waist-to-hip ratio in assessing visceral abdominal fat and subcutaneous adipose tissue [14, 15].

Due to the limitations of BMI and WC, a better method of assessing the association between abdominal obesity and lumbar disc degeneration is needed. However, few studies have explored the relationship between abdominal adiposity measured by MRI and disc degeneration. In this study, we explored the association between abdominal adipose tissue thickness determined using MRI and lumbar disc degeneration in a Chinese patient population.

## RESULTS

### Disc degeneration grade distributions by gender

As shown in Table 1, grade 1 and 2 lumbar disc degeneration were more common in men than in women (both *P* < 0.001), while grade 3 and 5 degeneration were more common in women than in men (both *P* < 0.01). Women had higher mean L1/L2, L2/L3, L3/L4, and L4/L5 disc degeneration grades than men (all *P* < 0.01). Furthermore, women had higher disc degeneration sum grades than men (*P* < 0.001).

**Table 1:** Distribution of lumbar disc degeneration according to gender

	Total	Male	Female	
	*N* = 2415	*N* = 1098	*N* = 1317	*P*
Grade 1, n (%)	15 (0.12%)	15 (0.27%)	0 (0%)	<0.001
Grade 2, n (%)	4668 (38.66%)	2305 (41.99%)	2346 (35.63%)	<0.001
Grade 3, n (%)	2922 (24.2%)	1247 (22.71%)	1675 (25.44%)	0.001
Grade 4, n (%)	1772 (14.67%)	803 (14.63%)	969 (14.72%)	0.89
Grade 5, n (%)	2024 (16.76%)	771 (14.04%)	1253 (19.03%)	<0.001
Grade 6, n (%)	518 (4.29%)	252 (4.59%)	266 (4.04%)	0.14
Grade 7, n (%)	147 (1.22%)	74 (1.35%)	73 (1.11%)	0.23
Grade 8, n (%)	9 (0.07%)	6 (0.11%)	3 (0.05%)	0.20
Mean grade of disc degeneration				
L1/2	2.46±0.82	2.41±0.83	2.51±0.82	0.006
L2/3	2.66±0.98	2.56±0.93	2.73±1.01	<0.001
L3/4	3.07±1.12	2.95±1.10	3.17±1.13	<0.001
L4/5	3.98±1.19	3.87±1.22	4.07±1.15	<0.001
L5/S1	4.21±1.45	4.19±1.43	4.23±1.47	0.53
Sum grades of disc degeneration	16.38±3.63	15.98±3.52	16.71±3.69	<0.001

Data are shown as mean ± SD or number (percentage).

### Adiposity diameters differed between men and women

As shown in Table 2, AD and SAD were higher in men than in women (both *P* < 0.001), while VST and DST were lower in men than in women (both *P* < 0.001).

**Table 2:** Characteristics of adiposity parameters

Variables	Total	Male	Female	
	*N* = 2415	*N* = 1098	*N* = 1317	*P*
Age, year	45.0 (38.0-55.0)	44.0 (36.0-53.0)	47.0 (39.0-56.0)	<0.001
AD, cm	92.4 (77.8-108.4)	97.5 (83.3-114.7)	88.0 (74.4-103.8)	<0.001
SAD, cm	185.6 (168.4-204.5)	193.4 (177.0-212.1)	179.0 (163.4-197.5)	<0.001
VST, cm	18.9 (14.1-25.4)	15.2 (11.5-19.1)	23.5 (18.1-29.1)	<0.001
DST, cm	15.1 (10.7-20.5)	12.3 (8.8-17.0)	17.5 (13.1-22.8)	<0.001

Data are shown as median (interquartile ranges).

Abbreviations: AD indicates abdominal diameter; SAD, sagittal diameter; VST, ventral subcutaneous thickness; DST, dorsal subcutaneous thickness.

### Associations between adiposity diameters and lumbar disc degeneration sum grades

As shown in Table 3, Spearman correlation analysis revealed that AD and SAD were positively correlated with lumbar disc degeneration sum grades in men (*r* = 0.120 and *r* = 0.131, respectively, both *P* < 0.001). Conversely, DST was negatively associated with lumbar disc degeneration sum grades in men (*r* = −0.079, *P* = 0.009). VST was not associated with lumbar disc degeneration sum grades in men. In women, AD, SAD and VST were positively correlated with lumbar disc degeneration sum grades (*r* = 0.294, *r* = 0.295 and *r* = 0.187, respectively, all *P* < 0.001). DST was not associated with lumbar disc degeneration sum grades in women.

**Table 3:** The associations between adiposity diameters and sum grades of lumbar disc degeneration

Sum grades of lumbar disc degeneration				
	Male		Female	
	*r*	*P*	*r*	*P*
AD, cm	0.120	<0.001	0.294	<0.001
SAD, cm	0.131	<0.001	0.295	<0.001
VST, cm	−0.025	0.41	0.187	<0.001
DST, cm	−0.079	0.009	−0.005	0.86

Abbreviations: r indicates spearman correlation coefficient; AD, abdominal diameter; SAD, sagittal diameter; VST, ventral subcutaneous thickness; DST, dorsal subcutaneous thickness.

### Associations between adiposity diameters and severe lumbar disc degeneration

As shown in Table 4, the crude model indicated that AD, SAD, and VST were positively associated with severe lumbar disc degeneration in men (all *P* < 0.05); men in the highest quartiles for AD, SAD, and VST were at higher odds ratio of severe lumbar disc degeneration than those in the lowest quartiles (odds ratio (OR) = 1.82, 95%CI = 1.29-2.57; OR = 2.04, 95%CI = 1.45-2.87; OR = 1.49, 95%CI = 1.06-2.09; respectively). After adjusting for age, men in the highest quartiles for AD, SAD, and VST were still at higher odds ratio of severe lumbar disc degeneration (OR = 1.55, 95%CI = 1.09-2.21; OR = 1.79, 95%CI = 1.26-2.55; OR = 1.77, 95%CI = 1.24-2.52; respectively). DST was not associated with severe lumbar disc degeneration in men in either model. In women, the crude model indicated that AD, SAD, and VST were positively associated with severe lumbar disc degeneration (all *P* < 0.01), and women in the highest quartile for AD, SAD, and VST were at higher odd ratio of severe lumbar disc degeneration than those in the lowest quartiles (OR = 2.49, 95%CI = 1.78-3.48; OR = 2.57, 95%CI = 1.83-3.61; OR = 1.60, 95%CI = 1.15-2.21; respectively). After adjusting for age, women in the highest quartiles for AD and SAD were still at higher odds ratio of severe lumbar disc degeneration (OR = 1.80, 95%CI = 1.26-2.56; OR = 1.94, 95%CI = 1.37-2.77; respectively). However, VST was not associated with severe lumbar disc degeneration in women in the adjusted model, and DST was not associated with severe lumbar disc degeneration in women in either model.

**Table 4:** Association analysis of adiposity diameters with severe lumbar disc degeneration

	Severe lumbar disc degeneration			
	Males		Females	
	Crude OR (95% CI)	Adjusted* (95% CI)	Crude OR (95% CI)	Adjusted* (95% CI)
AD quartiles				
Quartile 1	1.00	1.00	1.00	1.00
Quartile 2	1.42 (1.01-1.99)	1.25 (0.88-1.77)	1.17 (0.86-1.59)	1.08 (0.79-1.48)
Quartile 3	1.36 (0.97-2.25)	1.18 (0.83-1.67)	1.99 (1.43-2.75)	1.67 (1.19-2.33)
Quartile 4	1.82 (1.29-2.57)	1.55 (1.09-2.21)	2.49 (1.78-3.48)	1.80 (1.26-2.56)
*P* for trend	0.002	0.03	<0.001	<0.001
SAD quartiles				
Quartile 1	1.00	1.00	1.00	1.00
Quartile 2	1.89 (1.34-2.65)	1.70 (1.20-2.41)	1.20 (0.88-1.63)	1.05 (0.76-1.45)
Quartile 3	1.75 (1.25-2.46)	1.51 (1.06-2.14)	1.60 (1.16-2.20)	1.29 (0.93-1.80)
Quartile 4	2.04 (1.45-2.87)	1.79 (1.26-2.55)	2.57 (1.83-3.61)	1.94 (1.37-2.77)
*P* for trend	<0.001	0.004	<0.001	<0.001
VST quartiles				
Quartile 1	1.00	1.00	1.00	1.00
Quartile 2	1.40 (1.00-1.97)	1.39 (0.98-1.97)	1.12 (0.81-1.54)	1.02 (0.73-1.41)
Quartile 3	1.60 (1.14-2.25)	1.72 (1.21-2.44)	1.32 (0.96-1.82)	1.13 (0.81-1.57)
Quartile 4	1.49 (1.06-2.09)	1.77 (1.24-2.52)	1.60 (1.15-2.21)	1.29 (0.92-1.81)
*P* for trend	0.01	<0.001	0.003	0.11
DST quartiles				
Quartile 1	1.00	1.00	1.00	1.00
Quartile 2	1.01 (0.72-1.42)	1.05 (0.74-1.50)	0.79 (0.57-1.09)	0.80 (0.58-1.11)
Quartile 3	1.02 (0.72-1.43)	1.10 (0.77-1.56)	1.00 (0.72-1.39)	1.07 (0.76-1.49)
Quartile 4	0.98 (0.70-1.38)	1.13 (0.79-1.61)	0.94 (0.68-1.31)	1.03 (0.74-1.44)
*P* for trend	0.92	0.48	0.90	0.48

* Adjusted for age

Abbreviations: OR indicates odds ratio; CI, confidence interval; AD, abdominal diameter; SAD, sagittal diameter; VST, ventral subcutaneous thickness; DST, dorsal subcutaneous thickness.

## DISCUSSION

In this study, we found that increased AD and SAD were associated with a higher odds ratio of severe lumbar disc degeneration in both men and women, while high VST was associated with an increased odds ratio of severe lumbar disc degeneration only in men.

Disc degeneration is characterized by a reduction in signals of the nucleus pulposus and the inner fibers of the anulus [16]. Lumbar disc degeneration plays a key role in lower back pain [17]. Many factors, including genetic inheritance, early environment, age, loading history, and inadequate metabolite transport, can weaken discs and result in structural failure during routine daily activities [18–20]. In addition to the above factors, our findings suggest that increased abdominal adipose tissue thickness is associated with lumbar disc degeneration.

Previous research has revealed that high BMI is closely associated with an increased risk of lumbar disc degeneration. Like *et al*. gathered 4 years of follow-up data in a population-based MRI study and found that a BMI ≥ 25kg/m^2^ increases the risk of lumbar disc degeneration; patients who were overweight at young age were at particularly high risk [10]. Another population-based study performed by Samartzis *et al*. indicated that overweight or obese adults had higher rates of, more extensive, and more severe lumbar disc degeneration [3]. They also found that high BMI was strongly associated with the presence and severity of juvenile disc degeneration [6].

The above-mentioned studies all used BMI to measure body composition, despite its limitations. Adiposity parameters measured using MRI are strongly correlated with BMI and waist circumference (WC), and are superior to WC for assessing amounts of abdominal fat [14]. Recently, Jani *et al*. demonstrated that increased measures of abdominal obesity obtained using MRI are associated with increased lumbar disc degeneration in young men. However, they only measured AD and SAD, and did not identify a similar correlation in females. Our results are consistent with their findings in men. Furthermore, we identified the same correlation in women, and found that VST was also related to lumbar disc degeneration in men. Differences in patient age between the studies and in the ethnic groups examined, which are likely associated with differences in daily activities and fat distribution, might explain these discrepancies. Although the statistically significant regression coefficients in our study were relatively small, these associations might still have important implications for public policy and health care related to obesity.

Several possible mechanisms might contribute to the association between abdominal adipose tissue thickness and disc degeneration. Accumulation of abdominal fat might increase the mechanical load on the spine by increasing compressive forces and shear in lumbar structures during daily activity. People with high BMIs may also be more likely to experience accidental spinal injuries [8]. In addition, atherosclerosis is closely related to disc degeneration. Aortic atherosclerosis and stenosis of the feeding arteries in the lumbar spine reduce blood flow, resulting in disc degeneration and lower back pain [21]. Many well-known risk factors contribute to atherosclerosis, including hyperlipidemia, hyperglycemia, and hypertension [9]. Accumulation of abdominal fat is closely associated with these risk factors [22]. In addition, over-accumulation of abdominal fat causes chronic low-grade inflammation, which plays a crucial role in the pathogenesis of atherosclerosis [9, 23]. Adipose tissue is a dynamic endocrine organ that secretes several inflammatory mediators called adipocytokines, such as IL-6, IL-18, and TNF-a, which trigger production of C-reactive protein in the liver [9]. The accumulation of excess abdominal fat thus increases inflammation, which promotes endothelial dysfunction and atherosclerosis.

To the best of our knowledge, this is the first large-scale study to assess the relationship between abdominal adipose tissue thickness and lumbar disc degeneration in a Chinese patient population. The use of a 3T MRI system scanning with high resolution also helped to ensure accurate diagnoses. Finally, the modified Pfirrmann grading system used here allowed us to accurately determine the severity of disc degeneration in elderly subjects. However, several limitations of this study should be considered. First, as retrospective studies are associated with an unavoidable bias, prospective studies and multicenter validations should be performed. Second, we did not account for confounding factors aside from age and gender, such as body size, family history and lifestyle factors including diet, smoking, and physical activity levels; these factors should be considered in future studies. Third, overall body composition, including fat and fat-free mass, was not examined. Additional studies should incorporate these measures to more accurately account for the involvement of inflammation and the metabolic syndromes in disc degeneration. In addition, lower back pain was not assessed in this study.

In conclusion, we found that AD and SAD were associated with lumbar disc degeneration in both men and women, and VST was also related to lumbar disc degeneration in men, suggesting that abdominal obesity might be one of underlying mechanisms of lumbar disc degeneration. A longitudinal study would be particularly helpful for further investigating this relationship and its correlation with pain.

## MATERIALS AND METHODS

### Subjects

This retrospective study was conducted in participants selected from a total of 7541 patients who received lumbar spine MRI examinations for different reasons between November 2011 and November 2013 at The Second Affiliated Hospital of Wenzhou Medical University, China. Among these 7541 subjects, those who met the following criteria were excluded: 1) 4021 with unclear abdominal adipose tissue edges under the skin; 2) 628 with lumbar trauma history or spinal deformities; 3) 338 who previously underwent spinal surgery; and 4) 139 with benign or malignant spinal tumors. Ultimately, 2415 participants were included in this study. Demographic information, such as age and sex, were collected from the hospital database. The Institutional Review Board of The Second Affiliated Hospital of Wenzhou Medical University approved this study, and informed consent was obtained from all participants.

### Lumbar magnetic resonance imaging

All participants underwent a lumbar spine MRI scan on a single high field strength system (3.0 Tesla Signa HDxt EXCITE, General Electric, Milwaukee, USA) with a multichannel phased array spine surface coil, using a standardized Sagittal spin-echo T2 (TR 2500 ms, TE 107 ms), in a supine position. The field of view was 320×320 mm^2^, with an acquisition matrix of 256×256. The slice thickness was 4 mm, with a 1 mm inter-slice space, and signals were acquired twice.

### Disc degeneration assessment

We evaluated the degree of L1/2 to L5/S1 (L = lumbar, S = sacral) disc degeneration in T2-weighted lumbar spine MRI images using the modified Pfirrmann grading system, an 8-level system in which each grade represents a stepwise progression from normal disc (grade 1) to severe disc degeneration (grade 8) [16]. Degeneration was classified mainly based on the signal intensity of the nucleus pulposus and the inner fibers of anulus, changes in signal intensity between the inner and other posterior anulus fibers, and disc space height. Sum grades (theoretically ranging from 0 to 40, but from 0 to 30 in study subjects) of all five lumbar discs were calculated for each subject according to the individual disc grades. Two examiners were trained by the same experienced radiologist to perform disc degeneration assessments. Each examiner performed all anthropometric measurements independently with the aid of an illustrated anthropometry manual. Discrepancies were identified and reconciled by the radiologist.

### Anthropometric and adiposity measures

**Figure 1:**
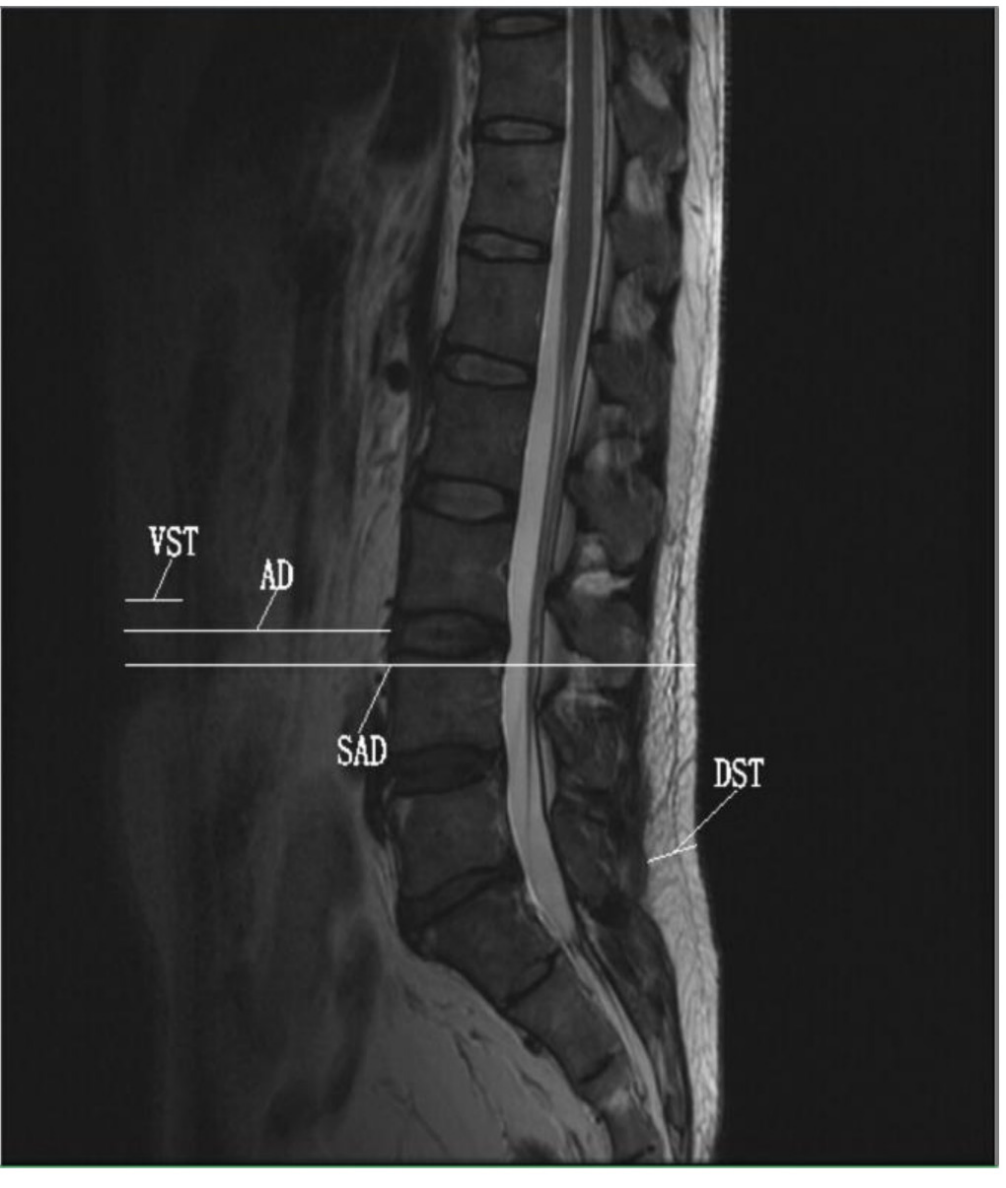
T2-weighted midsagittal plane images of the lumbar spine showing the adiposity diameters measured Abbreviations: VST, ventral subcutaneous thickness; AD, abdominal diameter; SAD, sagittal diameter; DST, dorsal subcutaneous thickness.

We measured adipose tissue diameters in four locations in the midsagittal plane from T2-weighted images. Sagittal diameter (SAD, cm) was defined as the horizontal distance between anterior skin and posterior skin at the umbilicus level [24]. Abdominal diameter (AD, cm) was defined as the distance from the abdominal subcutaneous fascia to the anterior border of the vertebral body at the same SAD level [25]. Ventral subcutaneous thickness (VST, cm) was the thickness of the subcutaneous adipose tissue at the same SAD and AD level [26]. Dorsal subcutaneous thickness (DST, cm) was defined as the distance between the anterior edge of the subcutaneous fat and the subcutaneous fascia in the L5 or S1 vertebral body perpendicular to the skin at the presacral level [15] (Figure 1).

### Statistical analysis

Statistical analysis was performed using SAS version 8.1 (SAS Institute, Cary, NC); a two-sided *P* value < 0.05 indicated a significantly significant difference. Data are presented as means ± SD, medians with interquartile ranges, or numbers with proportions for categorical variables. Study subjects were divided in two groups according to gender. Continuous variables and categorical variables were compared between groups using *t*-tests and χ^2^ tests, respectively. Spearman correlations were used to explore correlations between adiposity diameters and lumbar disc degeneration sum grades. Severe lumbar disc degeneration was defined by at least one grade ≥ 5 in the same subject. Adiposity parameters were divided into quartiles, with the first quartile representing the lowest 25% of patients and the fourth quartile representing the highest 25% of patients. Crude associations between adiposity parameters and severe lumbar disc degeneration were first assessed using univariate logistic regression, and multivariable logistic regressions were then performed to adjust for age effects in the model.
